# Physiotherapeutic assessment and management of chronic pelvic pain syndrome: A case report

**DOI:** 10.1097/MD.0000000000025525

**Published:** 2021-04-16

**Authors:** Bartlomiej Burzynski, Tomasz Jurys, Kamil Burzynski, Katarzyna Cempa, Andrzej Paradysz

**Affiliations:** a Department of Rehabilitation; b Doctoral School, Faculty of Health Sciences in Katowice, Medical University of Silesia, Katowice; c UROSILESIA Urological, Gynaecological and Proctological Centre of Physiotherapy, Zabrze; d Department of Urology, Faculty of Medical Sciences in Zabrze, Medical University of Silesia, Katowice, Poland.

**Keywords:** case report, chronic pelvic pain syndrome, physiotherapy techniques

## Abstract

**Introduction:**

Chronic pelvic pain syndrome is difficult for the diagnosis and therapy and that means the patient attending the physician or physiotherapist may present various symptoms. There are no guidelines concern physiotherapy diagnosis and treatment of chronic pelvic pain syndrome. This study presents the physiotherapeutic assessment and management in men with pelvic pain symptoms.

**Patient concerns:**

Forty-six-year-old man attended the physiotherapy consulting room due to symptoms of pain in the perineum, lower abdomen, urethra, and scrotum for a year. Earlier, the patient had consulted a urologist who made a diagnosis of cystitis and prescribed medications that did not get results.

**Diagnosis:**

Ultrasound imaging and manual inspection (per rectum) of the pelvic floor was conducted by physiotherapist. Also, the abdominal and lower extremities muscles were assessed. Patient reported pain symptoms during examination of the musculus ischiocavernosus, puboanalis, pubococcygeus, iliococcygeus, coccygeus, and canalis pudentalis seu Alcocki.

**Interventions:**

The patient was given physiotherapeutic interventions consisting in the manual therapy of the lumbopelvic hip complex and the manual therapy per rectum.

**Outcomes:**

During 10th session of the physiotherapeutic treatment, patient reported improvement in pain symptoms. A month later, patient reported total alleviation of the pain symptoms during control visit.

**Conclusion:**

Therapy of chronic pelvic pain syndrome is a process that involves application of different therapies and different approaches. Functional and structural assessment and also therapy conducted by physiotherapist is becoming an integral part of urology and represents 1 possible conservative treatment form.

## 1 Introduction

Chronic pelvic pain syndrome in males poses many challenges both diagnostic and therapeutic. Often, the primary diagnosis made by urologists indicates cystitis (International Statistical Classification of Diseases and Related Health Problems 10th revision [ICD10]: N30) and/or prostatitis (ICD10: N41).^[1]^ The most common symptoms of cystitis are pain during urination, pollakiuria, a constant feeling of bladder pressure, and foul-smelling, dark, or turbid urine. In the case of prostatitis, patients commonly report pain in the area of the pubic symphysis, perineum, and testicles after prolonged sitting. They may also experience pain in the lumbosacral part of the spine, a burning sensation in the urethra independent of micturition, or pollakiuria.^[2]^

The complexity of the diagnostic and therapeutic process means that patients attending physiotherapy may present a somewhat different pattern of symptoms and functions than that expected on the basis of ICD-10 diagnosis. Cooperation between urologist and physiotherapist is, therefore, necessary to ensure selection of effective methods and means of therapy.

## 2 Case report

On August 4, 2020, a 46-year-old man attended the physiotherapy consulting room due to symptoms of pain in the perineum, lower abdomen, urethra, and scrotum. The first symptoms appeared in December 2019.

Before his first visit, the patient had consulted a urologist on 2 occasions, reporting the abovementioned pain symptoms as well as a burning sensation in the urethra during urination. Urinalysis, kidney, and bladder ultrasound identified no irregularities. Following interview and tests, the urologist made a diagnosis of cystitis (ICD10: N30), for which the patient was prescribed ciprofloxacin (500 mg, 2 times per day, 5 days) and physiotherapeutic consultation. During the interview, it emerged that the patient was not sexually active due to the pain symptoms. In view of the back pain symptoms reported by patient, a magnetic resonance imaging scan was performed on July 20, 2020. This revealed features of discopathy at the L_5_/S_1_ level but with no nerve root compression. The patient reported no ongoing pharmacotherapy or treatment by other specialists. The previous treatment did not get results.

Structural and functional assessment of the pelvic floor muscles was conducted by means of both ultrasound imaging and manual inspection (palpation per rectum) during the first visit. In addition, the muscles of the anterolateral abdominal wall, the gluteal muscles, and the muscles of the lateral line and the back line of the lower limbs were assessed.

Functional diagnosis began with ultrasound assessment of bladder filling. This examination was performed using the Mindray Z5 Digital Ultrasonic Diagnostic Imaging System (Mindray Building, Keji 12th Road South, High-tech Industrial Park, Nanshan, Shenzhen 518057, P.R. China). A convex transducer (35C50EA, ibid.) was used in accordance with the methodology described by Tyloch and Wieczorek.^[3]^ Based on the measurements of the bladder collected, its volume was estimated at 240 mL. During the examination, the patient was asked to give subjective impressions of bladder pressure on a scale of 0 to 10 (0 = no feeling of bladder pressure, 10 = very strong micturition urgency). The patient gave a figure of 7. Following evaluation, the patient was asked to empty his bladder and the bladder volume assessment was then conducted again in order to verify urine retention. Assessment revealed no urine retention after urination in the present case.

Next, evaluation of volitional phasic contraction of the pelvic floor muscles was performed. The patient, without prior instruction, was asked to perform a rapid contraction of the pelvic floor muscles while the physiotherapist assessed the dynamics of contraction, its direction (elevation, depression, or no change), and the presence of possible peripheral stabilization (expressed as increased muscle tension of the abdominal, gluteal, and lower extremity muscles). The patient presented correct phasic contraction of the pelvic floor muscles (with elevation) with no apparent peripheral stabilization. For the purpose of assessing tonic activity, the patient was asked to perform a contraction of the pelvic floor muscles and maintain it for 8 seconds. The same aspects were assessed – that is, dynamics of contraction, direction, and presence of peripheral stabilization – but also ability to maintain contraction. The patient demonstrated correct tonic contraction of the pelvic floor muscles (with elevation) with no visible peripheral stabilization.

The physiotherapist next carried out per rectum examination of the patient in the position of lying on his side with knees flexed closer to thorax. The examination began with an observation of perineal area, during which the physiotherapist observed skin color and the appearance of the rectum, and checked for the existence of scar tissue and/or hemorrhoids. There were no visible pathological changes in the present case. The physiotherapist also assessed the flexibility of and prevalence of pain in the central tendon of the perineum by means of palpation. The patient did not report any painful symptoms and the central tendon of his perineum was flexible. In addition, after manual skin irritation, the correct anal reflex was observed.

The next step in the examination was palpation of the pelvic floor structures per rectum to determine level of tension and occurrence of pain. During palpation, the patient was asked to report any pain and define its intensity on a numeric rating scale (NRS) from 0 (= no pain) to 10 (= worst possible pain). The muscles were examined on both sides. Results are presented in Table 1.

**Table 1:** Pain assessment of pelvic floor muscles during palpation.

	Pain on NRS	
Examined structure	Left side	Right side
Prostate	0	0
Musculus ischiocavernosus	6	5
Musculus puboanalis	5	6
Musculus pubococcygeus	4	6
Musculus obturator internus	0	0
Musculus iliococcygeus	5	5
Musculus coccygeus	5	6
Canalis pudendalis seu Alcocki	6	5

After this, palpation assessment of phasic and tonic contraction of selected pelvic floor muscles was performed. The physiotherapist placed the index finger in the rectum in the musculus ischiocavernosus area and asked the patient to perform rapid contraction of the pelvic floor muscles (phasic contraction), during which ability to contract, contraction dynamic, and contraction strength were assessed. The physiotherapist then asked the patient to perform another contraction of the pelvic floor muscles and maintain it for 8 seconds (tonic contraction). Again ability to contract and the dynamics and strength of contraction were assessed. In every test, the patient presented correct reactions with no apparent peripheral stabilization.

In the next stage, examination of the lumbopelvic hip complex was carried out with the patient lying on his back and his lower extremities fully extended. The physiotherapist performed palpation assessment in the anterolateral abdominal wall area using both hands. Muscle tension and pain were evaluated. During palpation, the patient reported any pain and defined its intensity using the NRS. The physiotherapist assessed the following areas, with results presented in parentheses:

- musculus rectus abdominis at the level of umbilicus on the left side (0) and right side (0);
- musculus psoas major on the left side (0) and right side (0);
- musculus iliacus on the left side (0) and right side (4);
- musculus transversus abdominis in the middle of the line connecting the anterior superior iliac spine and public symphysis on the left side (5) and right side (5).

In the same position, the Lasèque test was employed. This test consists of slow raising of the straightened leg until onset of pain. During the test, the physiotherapist paid particular attention to the position of the pelvis (because of possible compensation) and the angle between leg and rest surface at the moment when pain occurred. The test was negative on both sides (the observed angle was >60°). The patient did not report any pain symptoms in the lumbosacral area or the lower extremities during the test.^[4]^ Thereafter, tension of the muscles of the back line and the lateral line in supine position was assessed. In order to evaluate the back line, the physiotherapist raised the patient's straightened leg with simultaneous dorsiflexion of the ankle. The leg was raised until resistance was encountered, signaled by a sensation of pulling along the course of the back line (indicated by the patient) or by apparent compensation in the form of knee flexion.^[5]^ In addition, the patient was asked to report any pain and define its intensity on the NRS. Along the course of the back line, the patient did not report pain symptoms on the left or right side. Examination of the lateral line was performed in the same position. The physiotherapist raised the patient's straightened leg with simultaneous dorsiflexion of the ankle and abducted hip joint. The leg was abducted until resistance was encountered, signaled by a sensation of pulling along the course of the lateral line (indicated by the patient) or by apparent compensation in the form of knee flexion.^[5]^ Along the course of the lateral line, the patient again did not report any pain on the left or right side

As the final stage of physiotherapeutic assessment, the musculus piriformis was evaluated. Again the patient remained in the same position as above. The physiotherapist, using both hands, performed musculus piriformis palpation along the course of the muscle and assessed muscle tension and pain. The patient was asked to report any pain and define its intensity on the NRS. Once again the patient did not report any pain on the left or right side.

Following physiotherapeutic evaluation and physician consulting, the diagnosis of chronic pelvic pain syndrome was made. Then, the patient was judged eligible for urologic physiotherapeutic treatment. Manual therapy of the lumbopelvic hip complex and manual therapy per rectum were used. Manual therapy of the lumbopelvic hip complex consisted in trigger point therapy, friction massage, and manual diaphragm release. Manual therapy per rectum used trigger point therapy, friction massage, and post-isometric relaxation. The course of physiotherapeutic treatment is presented in Table 2.

**Table 2:** Course of urogynecological physiotherapeutic treatment.

Number of session	Date of sessions	Therapies used	Duration of session
1	September 01, 2020	MT LPHC	40 min
2	September 03, 2020	MT LPHC	40 min
3	September 08, 2020	MT LPHC	40 min
4	September 10, 2020	MT LPHC	40 min
5	September 15, 2020	MT LPHC, MT PR	40 min
6	September 17, 2020	MT LPHC, MT PR	40 min
7	September 22, 2020	MT PR	40 min
8	September 24, 2020	MT PR	40 min
9	September 29, 2020	MT PR	40 min
10	October 01, 2020	MT PR	40 min

During the final session (October 01, 2020), the patient reported an improvement in relation to the pain symptoms presented.

On November 25, 2020, the patient attended a control visit during which he reported total alleviation of the pain symptoms. Additionally, the physiotherapist performed the entire evaluation once again, as during the first visit.

In ultrasound imaging (using a convex transducer), the bladder volume was estimated at 60 mL and patient did experience any sensation of bladder pressure. During examination, the patient presented correct phasic and tonic activity and elevation of the pelvic floor muscles. No retention of urine after urination was found. In the per rectum examination, the patient did not report any pain during palpation and showed correct phasic and tonic activity of the pelvic floor muscles. During palpation of the anterolateral abdominal wall area, the patient reported pain symptoms in each examined structure on the left side and defined its intensity as 1 or 2 on the NRS. On the right side pain symptoms were reported only during palpation of the musculus psoas major, the patient defining its intensity as 1.

The Lasèque test was negative on both sides. Palpation of the musculus piriformis did not trigger any pain symptoms. During examination of the back line and the lateral line the patient reported no painful symptoms.

The patient has provided informed consent for publication of the case.

## 3 Discussion

First-line treatment option includes analgesics, antibiotics, and α-adrenergic antagonists. Increasingly, the multidisciplinary approach including urologists, nurse specialists, physiotherapists, psychologists, sexual health specialists, etc is recommended.^[6]^ According to Katz et al, diagnoses made in collaboration between a larger number of specialists will tend to eliminate the possibility of diagnostic mistakes and may result in faster recovery times.^[7]^ Physiotherapy is one of the conservative treatment form in therapy of the chronic pelvic pain syndrome, which aim is a regulation of tension in the pelvic floor muscles and lumbopelvic hip complex, elimination of the myofascial trigger points. Moreover, the therapy of spinal and peripheral joints in the pelvis area is recommended.

This study presents an unusual approach connected with physiotherapeutic diagnosis. Using ultrasound imaging and assessing the pelvic floor muscle separately is not a standard procedure in daily practice.^[8,9]^ Currently, the diagnosis and therapeutic approach from present study shall not be extended to all patients suffering from chronic pelvic pain syndrome. It is associated with large individualization of physiotherapeutic assessment and management in the present case. Moreover, the present study could be of higher quality if the standardized questionnaire assessing the severity of symptoms has been used

Physiotherapeutic treatment aims to relieve symptoms and improve the quality of life of patients suffering from chronic pelvic pain syndrome. It should be noted that physiotherapeutic intervention, as a form of the conservative treatment, is an integral part of the entire process of treatment of urological disorders. Such physiotherapy may also be used to educate patients concerning lifestyle changes and/or techniques for autotherapy.^[10]^

## Acknowledgments

The authors greatly appreciate the assistance of Mr Alex Tilbury in the preparation of the English language version of this article. Mr Alex Tilbury has provided informed consent for publication of his name.

## Author contributions

**Conceptualization:** Tomasz Jurys, Kaml Burzynski, Katarzyna Cempa, Andrzej Paradysz, Bartlomiej Burzynski.

**Data curation:** Bartłomiej Burzynski, Tomasz Jurys, Kaml Burzynski, Katarzyna Cempa, Andrzej Paradysz.

**Formal analysis:** Bartłomiej Burzynski, Tomasz Jurys, Kaml Burzynski, Katarzyna Cempa, Andrzej Paradysz.

**Funding acquisition:** Bartłomiej Burzynski, Tomasz Jurys, Kaml Burzynski, Katarzyna Cempa, Andrzej Paradysz.

**Investigation:** Bartłomiej Burzynski, Tomasz Jurys, Kaml Burzynski, Katarzyna Cempa, Andrzej Paradysz.

**Methodology:** Bartłomiej Burzynski, Tomasz Jurys, Kaml Burzynski, Katarzyna Cempa, Andrzej Paradysz.

**Project administration:** Bartłomiej Burzynski, Tomasz Jurys, Kaml Burzynski, Katarzyna Cempa, Andrzej Paradysz.

**Resources:** Bartłomiej Burzynski, Tomasz Jurys, Kaml Burzynski, Katarzyna Cempa, Andrzej Paradysz.

**Software:** Bartłomiej Burzynski, Tomasz Jurys, Kaml Burzynski, Katarzyna Cempa, Andrzej Paradysz.

**Supervision:** Bartłomiej Burzynski, Tomasz Jurys, Kaml Burzynski, Katarzyna Cempa, Andrzej Paradysz.

**Validation:** Bartłomiej Burzynski, Tomasz Jurys, Kaml Burzynski, Katarzyna Cempa, Andrzej Paradysz.

**Visualization:** Bartłomiej Burzynski, Tomasz Jurys, Kaml Burzynski, Katarzyna Cempa, Andrzej Paradysz.

**Writing – original draft:** Bartłomiej Burzynski, Tomasz Jurys, Kaml Burzynski, Katarzyna Cempa, Andrzej Paradysz.

**Writing – review & editing:** Bartłomiej Burzynski, Tomasz Jurys, Kaml Burzynski, Katarzyna Cempa, Andrzej Paradysz.
